# The influence of skill and task complexity on perception of nested affordances

**DOI:** 10.3758/s13414-021-02355-5

**Published:** 2021-08-19

**Authors:** Ludovic Seifert, Matt Dicks, Frieder Wittmann, Peter Wolf

**Affiliations:** 1 University of Rouen Normandy, CETAPS lab. EA 3832, Rouen, France; 2 School of Sport, Health and Exercise Science, University of Portsmouth, Spinnaker Building, Cambridge Road, Portsmouth, PO1 2ER England; 3 Eidgenossische Technische Hochschule Zurich, Sensory-Motor Systems Lab, Zurich, Switzerland

**Keywords:** Perception, Action coupling, Affordances, Perceptual-motor control

## Abstract

This study investigated how skill level and task complexity influence the calibration of perception–action and particularly how close an individual acts relative to his or her maximal action capabilities. Complexity was manipulated between *two* (*Touch, Grasp*) and *more than two* (*Removing*, *Moving Up*) nested affordance conditions. For all conditions, we examined whether advanced climbers had greater maximal action capabilities than intermediate climbers or whether they better scaled their action (i.e., acted nearer to their maximal action capabilities) or both. Eleven intermediate and 11 advanced male climbers were first asked to estimate the maximum distance that they could reach a climbing hold. The hold was moved along a slide and fixed once requested by the participant; subsequently, the distance to the starting hold was measured. After each estimation, the participant was required to execute the climbing action. After four estimation-action trials in each of the four conditions, the maximal action capability (i.e., actual maximal reaching distance) was determined. Advanced climbers demonstrated greater actual maximal reaching distances than intermediate climbers for all conditions, but they only estimated greater maximal reaching distances for the more complex conditions, which featured *more than two *nested affordances. When estimated maximal reaching distances were scaled to actual maximal reaching distances, advanced climbers did not differ from intermediate climbers for any condition, and there were no differences between conditions. Our findings indicate that expertise was a function of greater action capabilities, but not due to the accuracy of calibration.

According to the ecological approach to visual perception (Gibson, 1979), perception and action are synergistic, comprising a mutual relationship between information pickup and human movements (Stoffregen, 2003). Gibson (1979) described opportunities for action offered by the environment as relative to action capabilities of an individual in terms of affordances. Contemporary perspectives have further emphasized that an individual must be able to perceive which action mode is possible among multiple affordances (Ye et al., 2009) and how to control one’s own movements in order to successfully achieve the intended task goal (Warren, 2006). The affordance-based control framework proposes that the development of perception and action requires perceptual attunement and the calibration of action (Fajen, 2005). Attunement relates to the pickup of more reliable information patterns in the energy arrays to guide action, while calibration concerns the appropriate scaling between information and an individual’s action capabilities (Fajen, 2005, 2007). Attunement and calibration are thought to support skilled action (Dicks et al., 2010), guiding the control of movements within a safe region that separate possible from impossible actions (Fajen & Devaney, 2006). The current study aims to further extant understanding regarding the calibration of perception–action and how skill and task demands might influence this feature of affordance-based control.

Affordances are nested over a number of different spatial and temporal scales (Wagman et al., 2016; Wagman & Morgan, 2010) and can be exploited sequentially, simultaneously or in parallel—that is, simultaneously but independently of one another (Mark et al., 2015). According to Wagman et al. (2016), nested affordances refer to the multiple affordances that exist in any given situation, and our consequent obligation to choose among them or to order them. Given that affordances reflect the relational nature of many properties of animals and the many properties of environments, affordances are proposed to be nested in the context of other affordances (Wagman & Stoffregen, 2020). The idea that affordances can be nested within each other implies that an individual affordance may be superordinate to other affordances and, therefore, that some affordances will be subordinate to others (Wagman & Stoffregen, 2020). Thus, Wagman et al. (2016) stated that a sequence of nested affordances emerges from the selection of a superordinate affordance, defining a subordinate/superordinate relationship between affordances.

Skilled athletic experience has been shown to influence the perception of nested affordances as a function of attunement (Boschker & Bakker, , 2002; Boschker et al., 2002). For instance, in climbing, skilled performers have been found to be better attuned to functional properties of holds (e.g., how best to grasp the hold with little effort and enable a high probability to move further) than less experienced climbers. Conversely, less experienced climbers have been found to exploit structural properties of holds such as shape, size, or texture, leading to a hold-by-hold climbing behaviour instead of chaining movements (Boschker & Bakker, 2002; Boschker et al., 2002). Chaining movements require specific bodily adaptations (e.g., rolling the body from one side to the other side) to the complexity of the route design of the climbing wall depending on the orientation of the holds (Seifert et al., 2015; Seifert et al., 2018), allowing multiple holds to be collectively perceived as a single nested climbing opportunity (Boschker et al., 2002; Seifert et al., 2017). However, the effect of task complexity comprising multiple nested affordances (i.e., when *more than two* nested affordances are chained) in relation to action capabilities has rarely been investigated (notably, following an ecological approach to perception, as highlighted by Whitaker et al., 2020) and therefore, current understanding remains unclear (Mark et al., 2015; Ye et al., 2009). A recent study of expert climbers has shown that during the completion of complex tasks comprising *more than two* nested affordances (e.g., reach to grasp a climbing hold with one hand before *Removing* the other hand from the starting hold; reach to grasp a climbing hold with one hand, before *Moving up* to grasp another hold), coordination of the limbs and/or the distribution of forces between limbs, arms, and feet could be realized through various means during a climb, suggesting functional equivalence (Seifert et al., 2021). Accordingly, performance in conditions comprising *more than two* nested affordances were better explained by action capabilities than by body-scaled metrics. In contrast, during simpler reaching conditions involving *two* nested affordances (e.g., reach to touch and reach to grasp), estimated maximal reaching distance was found to relate to action-scaling and also to arm length (Seifert et al., 2021).

The evidence considered in the previous paragraph suggests that as climbers develop their expertise, they become better attuned to information that supports the perception of nested affordances (Seifert et al., 2021; Seifert et al., 2018). However, despite a growing body of literature that has examined attunement as a facet of affordance perception (for review, see Fajen, 2005; Fajen et al., 2009), few studies have investigated expertise relative to calibration (Higuchi et al., 2011; Hove et al., 2006; Weast et al., 2011). Higuchi et al. (2011) examined how groups of athletes with varying levels of competitive experience in different sports (American football, rugby, and control athletes) adapted their locomotion when moving between two ‘tackling’ dummies during a collision avoidance task. When running between the two dummies, the American football players exhibited a later onset and a smaller magnitude of shoulder rotations than control athletes, which contributed to a faster running speed, whereas no group differences were observed in a walking condition (Higuchi et al., 2011). These findings offer evidence that skill levels were differentiated due to the calibration of the onset of shoulder rotations relative to the tackling dummies. Further research is needed to understand action-scaling effects as a facet of expertise in affordance-based control (Dicks et al., 2019). In particular, a pertinent theoretical question in the study of expertise with regard to the theory of affordances is to understand whether skilled athletes have greater maximal action capabilities than less-skilled athletes or whether skilled athletes act nearer to their maximal action capabilities (i.e., they better scale their actions), or whether both facets contribute to expertise differences.

Several studies that have examined reaching and grasping have emphasized that affordances are scaled to maximal action capabilities (Pepping & Li, 2000; Wagman & Morgan, 2010), especially when reaching is goal-orientated and combines nested actions, such as reach to touch, reach to grasp, jump to reach (Pepping & Li, 2008; Ramenzoni et al., 2010; Wagman et al., 2019), and reach to climb (Croft et al., 2018; Pijpers et al., 2007; Pijpers et al., 2006). Nonclimbers have been shown to estimate their maximal overhead reaching capabilities less accurately than climbers (Pijpers & Bakker, 1993). Thus, one possibility is that skilled performers better scale their actions relative to their maximal action capabilities in comparison with less-skilled athletes, thus enabling experts to act closer to their affordance boundaries (Fajen, 2005). Alternatively, it is possible that both skilled and less-skilled athletes are equally well calibrated, but that differences in the maximal action capabilities of the respective skill levels present different opportunities for action (Dicks et al., 2010).

The literature considered has highlighted that there is limited current understanding regarding expertise effects during the perception of nested affordances of differing complexity and the calibration of perception–action relative to these varying task demands. Accordingly, the aim of the current study was to better understand the calibration of perception–action by examining how the estimation of maximal reaching distance relates to climbing expertise and task complexity. We investigated whether advanced climbers have greater maximal action capabilities (MAC), than intermediate climbers or whether they better scale their actions (i.e., act nearer to their MAC), reflected by a ratio closer to 1 between estimated maximal reaching distance and MAC, or both. Maximal action capabilities corresponding to the actual maximal reaching distance were assessed by incrementally increasing the distance between the hold and the participant until the climber could no longer reach/climb using the hold without falling (more details are provided in the method section). Intermediate and advanced climbers (according to the definition of Draper et al., 2011) had to master reaching conditions with *two* nested affordances (Condition 1: reach to *Touch* a climbing hold; and Condition 2: reach to *Grasp* a climbing hold) and reaching conditions with *more than two* nested affordances (Condition 3: reach to grasp a climbing hold with one hand before *Removing* the other hand from the starting hold; and Condition 4: reach to grasp a climbing hold with one hand, before *Moving up* to grasp another hold). First, we expected that advanced climbers have greater MAC than intermediate climbers, a finding that would be particularly evident in conditions of greater complexity (i.e., when *more than two* nested affordances are present). Second, we expected that advanced climbers would be better calibrated than intermediate climbers, and therefore act nearer to their MAC and with greater success (i.e., with a lower number of fails, lower overestimation) than intermediate climbers, an observation that would also be most prevalent in the conditions of greater complexity.

## Method

### Participants

Twenty-two male volunteers participated in the study and were split into two groups. Eleven climbers (age: *M* = 29.5 years, *SD* = 4.4 years; height: *M* = 177.5 cm, *SD* = 4.8 cm; arm span: *M* = 182.5 cm, *SD* = 6.2 cm; arm length: *M* = 74.6 cm, *SD* = 3.6 cm; leg length: *M* = 98.3 cm, *SD* = 7.2 cm; weight: *M* = 70.2 kg, *SD* = 6.3 kg) had a climbing ability of between 6c+ and 7b when lead climbing on sight. That is, with no prior knowledge of the route, on the French Rating Scale of Difficulty (Delignières et al., 1993), which represents an advanced level of performance (Draper et al., 2011). Ten out of the 11 advanced participant data sets have already been presented before (Seifert et al., 2021). Those participants practice indoor climbing: *M* = 75.9% of their time of practice, *SD* = 18.9%, consisting mainly in bouldering: *M* = 69.1%, *SD* = 18.1%. of their time of practice.

Eleven climbers (age: *M* = 28.8 years, *SD* = 4.9 years; height: *M* = 178.7 cm, *SD* = 5.8 cm; arm span: *M* = 183.2 cm, *SD* = 7.4 cm; arm length: *M* = 74.6 cm, *SD* = 3.0 cm; leg length: *M* = 100.7 cm, *SD* = 8.8 cm; weight: *M* = 72.1 kg, *SD* = 5.7 kg) had a climbing ability of between 6a and 6b when lead climbing on sight (i.e., with no prior knowledge of the route) on the French Rating Scale of Difficulty, which represents an intermediate level of performance (Draper et al., 2011). Those participants practice indoor climbing: *M* = 79.4% of their time of practice, *SD* = 16.9%, consisting mainly in bouldering: *M* = 73.1%, *SD* = 18.4% of their time of practice.

*T* tests confirmed that advanced climbers engaged in significantly more practice in climbing (*M* = 7.7 hours per week, *SD* = 3.9 hours per week), *t*(20) = 2.61, *p* = .017, and significantly more climbing experience (*M* = 11.0 years, *SD* = 4.4 years), *t*(20) = 3.28, *p* = .004, than intermediate climbers (*M* = 4.3 practice hours per week, *SD* = 1.3 hours per week and *M* = 5.4 years of practice, *SD* = 3.4 years of practice), but did not differ in terms of anthropometric characteristics.

As previous studies observed anthropometric, strength (i.e., bent-arm hang time and grip strength), flexibility and climbing ability differences between males and females (Baláš et al., 2012; Grant et al., 2001; Mermier et al., 2000; Watts et al., 1993), participants of only one sex have been recruited in this study.

All participants gave their written informed consent prior to taking part in the experiment. The protocol was approved by the ETH Zurich Institutional Review Board (ID: ETH 2017-N-48) in accordance with the Declaration of Helsinki. The sample size was determined by using G*Power (Version 3.1.9.6, 2020; https://www.psychologie.hhu.de/arbeitsgruppen/allgemeine-psychologie-und-arbeitspsychologie/gpower.html) and following the recommendation of Faul et al. (2007). In order to run a mixed design analysis of variance (ANOVA), with two groups of different skill level (between-subjects variable) and four climbing tasks (within-subjects variable), with α = .05, power = .80, effect size = .30 (which represents the threshold for medium effect size; Cohen, 1988), G*Power recommended a total sample size of *n* = 18, which is smaller than our sample size. The estimated sample size of 18 participants was dependent on the assumption of a rather strong correlation of the outcomes in the different task conditions. Medium effect size was selected as skill effect would mainly occur in conditions involving *more than two* nested affordances while in conditions involving *two* nested affordances, skill effect might be less dominant as a previous study highlighted that reaching distance could be scaled to arm length, with a positive correlation (*r* = .70) between reaching distance and arm span for the reach to grasp condition (Seifert et al., 2021).

### Procedure

Prior to the experiment, participants undertook a self-regulated warm-up on a bouldering-climbing wall that was positioned next to the experiment. The wall was inclined by 10° from vertical, to ensure that the protocol was conducted on a slightly overhanging wall, which reduced any possibility that in the event of a fall, the participant could collide with anything, such as a hold (a hold is a feature of the climbing wall that is held or has the possibility of being held). The starting handhold was configured with a large undercut, which could be easily grasped by both hands. The starting foothold was a round shaped bar. The handhold that could be moved was a crimp (this ensured that at least, the first phalanx of each finger could be placed on it), the additional foothold was similar in shape to the first foothold. A familiarization with the starting holds was allowed before starting the protocol.

**Fig. 1:**
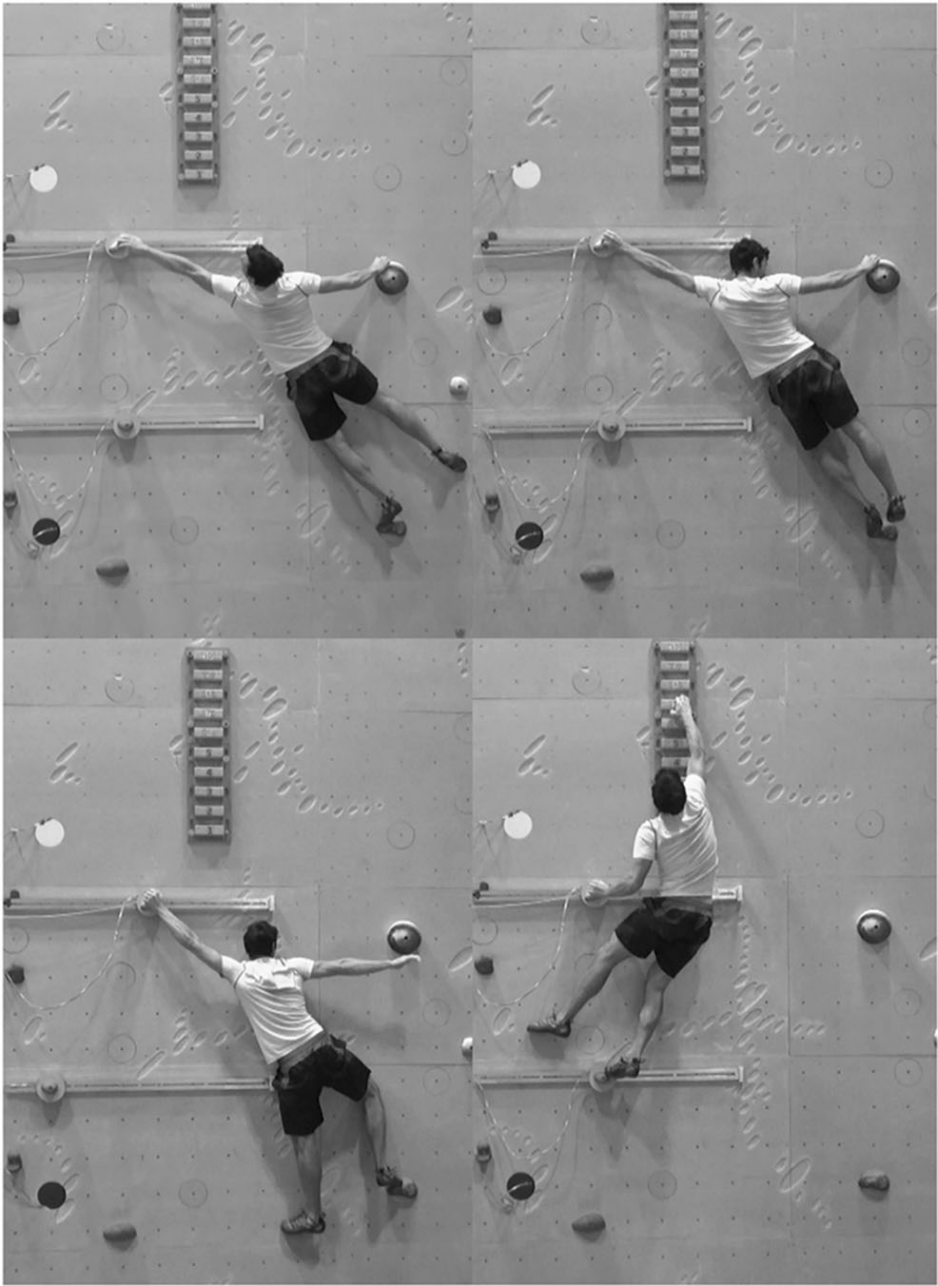
Final position in *Touch* condition (top left), in *Grasp* condition (top right), in *Remove* condition (bottom left), and in *Move Up* condition (bottom right)

**Fig. 2:**
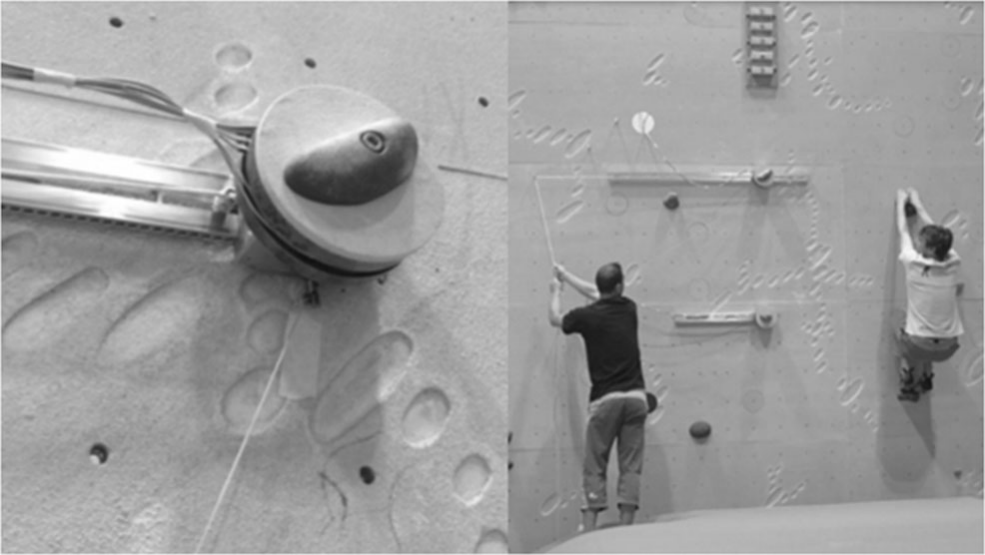
ledge mechanism to slide the targeted hold (left) and start position for distance estimation (right)

The protocol comprised of four different experimental conditions: *Touch, Grasp*, *Remove,* and *Move Up* (see Table 1 and Fig. 1). The first three conditions comprised of two phases. First, each participant was required to estimate his maximum capability to perform the required action. During estimation, the participant adopted a standardized starting position, which consisted of grasping the handhold with both hands, arms extended, and both feet on the foothold. Once the participant was in the start position, an experimenter moved the hold along a slide until the participant indicated the distance he may maximally master (see Table 1 and Fig. 2). During the motion of the hold along the slide, the participant remained stationary in the standardized starting position. Once the estimation had been made, the participant climbed down from the wall and had a short rest period while the hold was fixed in place. Thereafter, the participant was required to act in order to examine whether he was able to achieve the estimation (the participant performed the action after they had returned back to the start position). This sequence, estimation followed by action, was repeated for four trials in each condition.

**Table 1:** Overview on conditions

Condition	Estimation Description	Action Description
*Touch*(4 trials)	Remain in the starting position. Estimate the maximal distance that the hold can be reached and touched at the screw (i.e., centre of the hold) without falling. One foot and one hand must remain on the respective starting holds.	Reach and touch the screw (i.e., centre of the hold) for three seconds by one hand. Keep one foot and one hand on the respective starting holds.
*Grasp*(4 trials)	Remain in the starting position. Estimate the maximal distance that the hold can be grasped without falling. One foot and one hand must remain on the respective starting holds.	Reach and grasp the hold for three seconds. Keep one foot and one hand on the respective starting holds.
*Remove* (4 trials)	Remain in the starting position. Estimate the maximal distance that the hold can be grasped without falling. One foot and one hand must remain on the respective starting holds. One hand will grasp the target hold and the other hand will be removed.	Grasp the hold with one hand and remove the other hand from the starting hold for three seconds. Keep one foot on the starting foot-hold.
*Move Up maximum*(4 trials)		Grasp the highest possible hold on the wooden board for three seconds. Use the hand-hold and foot-hold on the slide to support your climb.
*Move Up* (4 trials)	Remain in the starting position. Estimate the maximal distance that the hand-hold can be used to move up without falling. A second foot-hold is available below the moved hand-hold and you will be required to grasp one bar below your maximum on the wooden board.	Grasp the hold with one hand and move up to grasp the wooden board with the other hand one bar below your previous maximum for three seconds without falling.

The protocol for the *Move Up* condition followed a slightly different protocol: first, participants were asked to grasp the highest hold possible on the wooden board (in order to determine their *Actual Maximal Move Up*; see Table 1), beginning the climb from the standardized staring position. Following the establishment of the participant’s maximal hold height, the same estimation followed by action sequence was carried out. The target hold on the wooden panel was set at one position below the highest hold. The second foothold was aligned to the moved handhold before the participant attempted to master the task.

Two different orders of the conditions were applied (random but counterbalanced assignment): Half of the participants were assigned to the order *Touch–Move Up–Grasp–Remove,* the other half to the order *Grasp–Remove–Move Up–Touch*. In total, each participant performed 16 estimations (four times for each condition) and 16 actions (for times for each condition). The resting time allowed between estimation and action was 1 minute, whilst the resting time between action and the next estimation condition was 4 minutes. No additional resting time was given between blocks of condition. During the 4 minutes resting, the climbers turned their back to the boulder wall and participated in a distraction task (i.e., they played a video game to not think about climbing). The total time for each participant to complete the entire protocol did not exceed two hours, which corresponds to the average climbing time a training session.

### Assessment of actual maximal reaching distance (maximal action capability; MAC)

A further test of the participant’s actual maximal reaching distance was carried out for each condition once the sequence of trials had been completed. Starting with the furthest estimated distance that was mastered, the distance of the hold from the participant was increased incrementally by a magnitude of 4%. This procedure was repeated until the participant was no longer able to complete the action for a given condition; when the participant was not able to master the distance, the failed distance was decreased by a magnitude of 2%. In the case that the participant was then able to master this distance, this distance was established as the maximum. If the distance was not mastered, then the prior mastered distance was the maximum. Between each attempt, as the hold was moved along the slide, the participant remained standing on the ground.

### Data collection

The distance the hold was moved on the slide was recorded by a tape measure, which was fixed at one-centimetre intervals. Success and failure were categorized during data collection and later confirmed using video footage recorded at 100 Hz.

### Data analysis and statistics

The task was achieved successfully (i.e., mastered) when the handhold was touched (for *Touch* condition) or grasped (for other conditions) with at least three fingers for three seconds. First, the estimated maximal reaching distance and the actual maximal reaching distance (i.e., maximal action capabilities; MAC), were recorded to determine whether advanced climbers had greater action capabilities than intermediate climbers and whether participants demonstrated lower action capabilities in more complex tasks. Second, the estimated maximal reaching distance was scaled to the MAC to determine whether advanced climbers better scaled their actions than intermediate climbers and whether participants demonstrated less accurate calibration in more complex tasks. A value of 1 would indicate that estimated maximal reaching distance equals exactly the actual MAC. A value greater than 1 would indicate an overestimation, which resulted in a fail (i.e., falling). A value less than 1 would indicate an underestimation. This action-scaled ratio was computed for each trial.

Third, for these metrics, we considered the best successful trial and the highest overestimation (i.e., falling) to understand how climbers act relative to their MAC. Given that behavioural stability is a feature of expertise, we also considered the range between the lowest underestimation and the highest overestimation to inform on the intertrial variability.

Homogeneity of variance (using Levene test) and normality of distribution (using Shapiro–Wilk test) were checked for inter-subject effect (i.e., skill level) before performing mixed-design ANOVAs, 2 skill levels (advanced, intermediate) × 4 tasks (*Touch*, *Grasp*, *Remove*, *Move Up*) to test skill and task effects on performance outcome (success vs*.* fail), the estimated maximal reaching distance, the actual maximal reaching distance (i.e., maximal action capability; MAC), the estimated maximal reaching distance when scaled to actual MAC, the highest overestimation and the range between the lowest underestimation and the highest overestimation. Sphericity in the repeated-measures design was verified with the Mauchly test (Winter et al., 2001). When the assumption of sphericity was not met, the significance levels of *F* ratios were adjusted according to the Greenhouse–Geisser procedure. Last, post hoc pairwise conditions comparison tests were applied and family-wise error rate was controlled by applying a Bonferroni correction of the *p* value (Howell, 2002). Partial eta squared (η_P_^2^) statistics were calculated as an indicator of effect size, considering that η_P_^2^ < .3 represents a small effect, .3 < η_P_^2^ < .5 represents a medium effect and η_P_^2^ > .5 represents a large effect (Cohen, 1988). All tests were performed using IBM SPSS Statistics 20.0 (1989-2011), with a level of statistical significance fixed at *p* < .05.

## Results

### Performance outcome (success vs. fail)

#### Skill level effect

Across all conditions, the mixed-design ANOVA did not reveal any significant effect of skill level, *F*(1, 20) = 1.93, *p* = .18, η_P_^2^ = .09, *β* = .26, meaning that intermediate climbers did not fail significantly more than advanced climbers. On a total of 176 trials, 55 fails occurred in intermediate climbers (i.e., 31.3% of all trials were not successful) and 41 fails occurred in advanced climbers (i.e., 23.3%).

#### Task complexity effect

Across all participants, the mixed-design ANOVA showed significantly greater fails when the tasks were more complex, *F*(3, 60) = 4.33, *p* = .011, η_P_^2^ = .18, *β* = .84. A pairwise test with Bonferroni corrections revealed that climbers failed more often in the *Remove* (*M* = 1.3, *SD* = 0.7 fails per participant and a total of 28 fails for the whole sample) and *Move Up* (*M* = 1.4, *SD* = 0.8 fails per participant and a total of 30 fails for the whole sample) conditions than in the *Grasp* (*M* = 0.6, *SD* = 0.7 fails per participant and a total of 14 fails for the whole sample) conditions, *F*(3, 18) = 4.14, *p* = .021, η_P_^2^ = .41, *β* = 0.76, whereas no significant differences were observed in the *Touch* condition (*M* = 1.1, *SD* = 0.7 fails per participant and a total of 24 fails for the whole sample).

### Effects of skill level and task complexity on the best estimated reaching distance

### Skill level effect

Across all conditions, when the *best successful trial* was considered, the mixed design ANOVA showed significant differences of estimated maximal reaching distance between groups, *F*(1, 20) = 15.74, *p* = .001, η_P_^2^ = .44, *β* = .96. Moreover, the mixed-design ANOVA also revealed significant interaction between task and skill, *F*(3, 60) = 6.35, *p* = .001, η_P_^2^ = .24, *β* = .93. A pairwise test with Bonferroni corrections revealed that advanced climbers demonstrated greater estimated maximal reaching distance than intermediate climbers in *Remove, F*(1, 20) = 26.11, *p* < .001, η_P_^2^ = .57, *β* = .99, and *Move Up. F*(1, 20) = 7.63, *p* = .012, η_P_^2^ = .21, *β* = .75, conditions (see Table 2).

**Table 2:** *Actual* and *best*-*estimated/mastered* maximal reaching distance for the different skill levels and conditions

Groups	Conditions	Actual maximal reaching distance (in cm)				Best estimated/mastered maximal reaching distance (in cm)			
		*M*	*SD*	CI inf	CI sup	*M*	*SD*	CI inf	CI sup
Advanced	*Touch*	167.8 *	3.8	162.8	172.8	164.7 *	5.8	159.9	169.4
	*Grasp*	172.1 *	5.8	167.0	177.1	168.3 *	8.2	161.9	174.6
	*Remove*	155.4 £	7.3	149.6	161.1	151.9 £	6.5	145.4	158.5
	*Move Up*	165.2 * £	7.1	159.6	170.9	161.5 * £	9.2	155.7	167.3
Intermediate	*Touch*	162.2 *	10.5	157.2	167.1	157.4 *	9.4	152.6	162.1
	*Grasp*	168.7 *	9.8	163.6	173.7	164.5 * **	11.5	158.2	170.8
	*Remove*	133.7	10.6	127.9	139.5	129.1	13.2	122.5	135.7
	*Move Up*	159.0 *	10.5	153.4	164.6	150.6 *	9.3	144.8	156.4
Total	*Touch*	165.0 *	8.2	161.5	168.5	161.0 *	8.2	157.7	164.4
	*Grasp*	170.3 *	8.1	166.8	173.9	166.4 * **	10.0	161.9	170.9
	*Remove*	144.5	14.2	140.5	148.6	140.5	15.5	135.9	145.2
	*Move Up*	162.1 *	9.3	158.2	166.1	156.1 *	10.6	151.9	160.2

*Note. M* = mean; *SD* = standard deviation; CI = confidence interval; * = significantly different to *Remove*; ** = significantly different to *Move Up*; £ = significantly different to intermediate climbers.

### Task complexity effect

Across all participants, when the *best successful trial* was considered, the mixed-design ANOVA revealed significant differences of estimated maximal reaching distance between conditions, *F*(3, 60) = 49.95, *p* < .001, η_P_^2^ = .70, *β* = 1. Moreover, the mixed design ANOVA revealed significant interaction between task and skill effect, *F*(3, 60) = 6.35, *p* = .001, η_P_^2^ = .24, *β* = .93. In advanced climbers, pairwise tests with Bonferroni corrections revealed that estimated maximal reaching distance was greater in *Touch* (*p* = .007) and *Grasp* (*p* < .004) than in *Remove* conditions, *F*(3, 18) = 5.18, *p* = .009, η_P_^2^ = .46, *β* = .86 (see Table 2). In intermediate climbers, pairwise tests with Bonferroni corrections revealed that estimated maximal reaching distance was greater in *Touch* (*p* < .001), *Grasp* (*p* < .001), and *Move Up* (*p* < .001) conditions than in *Remove* condition; estimated maximal reaching distance in *Grasp* condition was also significantly greater than in *Move Up* condition (*p* = .003), *F*(3, 18) = 24.77, *p* < .001, η_P_^2^ = .80, *β* = 1 (see Table 2).

### Effects of skill level and task complexity on maximal action capabilities

#### Skill level effect

The mixed-design ANOVA revealed significant differences of actual maximal reaching distance (i.e., maximal action capabilities, MAC) between groups, *F*(1, 20) = 11.91, *p* = .003, η_P_^2^ = .37, *β* = 0.91, but also significant interaction between skill level and task effects, *F*(3,60) = 8.63, *p* < .001, η_P_^2^ = .30, *β* = .99 (see Table 2). Pairwise tests with Bonferroni corrections revealed that advanced climbers had significantly greater MAC for the *Remove, F*(1, 20) = 30.91, *p* < .001, η_P_^2^ = .61, *β* = 1, and *Move Up, F*(1, 20) = 8.98, *p* = .01, η_P_^2^ = .49, *β* = .87, conditions than intermediate climbers (see Table 2).

#### Task complexity effect

Across all participants, the mixed design ANOVA revealed significant differences of MAC between conditions, *F*(3, 60) = 61.50, *p* < .001, η_P_^2^ = .75 *β* = 1. Pairwise test with Bonferroni corrections revealed that distances reached in *Touch*, *Grasp,* and *Move Up* conditions were greater than in the *Remove* condition (all *p*s = .001), *F*(3, 18) = 38.53, *p* < .001, η_P_^2^ = .86, *β* = 1 (see Table 2).

### Effects of skill level and task complexity on estimated maximal reaching distance when scaled to MAC

#### Skill level effect

When estimated maximal reaching distance was scaled to MAC, the mixed-design ANOVA did not reveal any significant effect of skill level on the *best successful trial, F*(1, 20) = 1.24, *p* = .27, η_P_^2^ = .06, *β* = .18, the *highest overestimation, F*(1, 20) = 1.24, *p* = .27, η_P_^2^ = .06, *β* = .18, and the *range between the lowest underestimation and the highest overestimation, F*(1, 20) = 1.51, *p* = .23, η_P_^2^ = .07, *β* = .21 (see Table 3).

**Table 3:** Effect of skill level and climbing task on the ratio between estimated maximal reaching distance and MAC, computed *on the best successful trial* (i.e., without falling), *the highest overestimation* (i.e., with falling) and the *range between the lowest underestimation and the highest overestimation*

Groups	Conditions	Best successful trial		Highest over-estimation		Range between lowest underestimation and highest overestimation	
		*M*	*SD*	*M*	*M*	*M*	*SD*
Advanced	*Touch*	0.98	0.02	1.01	0.03	0.12	0.06
	*Grasp*	0.97	0.02	1.00	0.02	0.14	0.06
	*Remove*	0.97	0.02	1.01	0.04	0.15	0.06
	*Move Up*	0.97	0.03	1.00	0.03	0.11	0.03
Intermediate	*Touch*	0.97	0.03	1.02	0.05	0.19	0.09
	*Grasp*	0.97	0.03	1.00	0.04	0.14	0.09
	*Remove*	0.96	0.05	1.05	0.05	0.13	0.08
	*Move Up*	0.95	0.06	1.01	0.04	0.16	0.10
Total	*Touch*	0.97	0.03	1.02	0.04	0.15	0.09
	*Grasp*	0.97	0.02	1.00	0.03	0.14	0.08
	*Remove*	0.97	0.03	1.03	0.05	0.14	0.07
	*Move Up*	0.96	0.05	1.00	0.04	0.14	0.08

*Note. M*= mean; *SD* = standard deviation.

#### Task complexity effect

The mixed design ANOVA did not reveal any significant effect of task complexity on the *best successful trial, F*(3, 60) = 0.61, *p* = .61, η_P_^2^ = .02, *β* = .16, the *highest over-estimation, F*(3, 60) = 2.64, *p* = .057, η_P_^2^ = .18, *β* = .62, and the *range between the lowest underestimation and the highest overestimation, F*(3, 60) = 0.22, *p* = .87, η_P_^2^ = .01, *β* = .09 (see Table 3).

## Discussion

The current experiment aimed to extend current understanding on the calibration of perception–action by examining how the estimation of maximal reaching distance relates to climbing expertise and task complexity. We investigated whether advanced climbers demonstrate greater MAC than intermediate climbers or whether they act nearer to their MAC (reflected by a ratio closer to 1 between estimated maximal reaching distance and MAC), or both, in conditions that comprised *two* and *more than two* nested affordances. As hypothesized, the current study showed that for the *more than two* nested affordance conditions (Condition 3: *Remove* and Condition 4: *Move Up*), advanced climbers had greater MAC and greater estimated/mastered maximal reaching distances than intermediate climbers. However, in contrast to our hypothesis, advanced climbers did not differ from intermediate climbers for any condition when estimated maximal reaching distance was scaled to MAC. Our findings also showed that (1) *Remove* and *Move Up* conditions were the most complex tasks for intermediate climbers, while the *Remove* condition indicated the lowest estimated maximal reaching distance for advanced climbers; (2) for both groups, MAC was greater for *Touch*, *Grasp* and *Move Up* conditions compared with *Remove* condition; (3) when scaled to MAC, estimated maximal reaching distance did not show an effect of condition for both groups.

According to this summary, the hypothesis that skill influenced the calibration of perception–action, especially when *more than two* nested behaviours were required, was partially validated. Although advanced climbers had greater MAC across all conditions and had greater estimated maximal reaching distances in conditions involving *more than two* nested affordances, they did not act nearer to their MAC than intermediate climbers. Thus, our findings emphasized that irrespective of skill level, climbers were calibrated and therefore affordance perception was scaled to MAC (see Table 3). This finding supports previous research in reaching and grasping, which demonstrates that affordances are scaled to MAC (Pepping & Li, 2000; Wagman & Morgan, 2010), especially when reaching is goal-orientated and combines nested actions (Croft et al., 2018; Pepping & Li, 2008; Wagman et al., 2019). Because calibration of perception–action did not differ with skill level—as also shown for a basic overhead reaching task within climbers (Whitaker et al., 2020)—our study suggests that to improve climbing skill from an intermediate to an advanced level and to perceive new opportunities for action, MAC may need to be enhanced (for similar suggestion, see Knobelsdorff et al., 2020).

The hypothesis that task complexity influenced the calibration of perception–action, notably with an interaction between task complexity and skill was also partially supported. Complex tasks involving *more than two* nested affordances like the *Remove* condition required a contralateral movement, as the climbers had to spread the forces between the left hand and the right foot, and control the balance of their body by applying friction with their left foot on the wall, in order to counterbalance the twist of the trunk. This movement required climbing specific muscle strength, which we did not assess in the current study. Since there is a high correlation between climbing specific muscle forces, such as maximum finger forces, and climbing level (Baláš, 2016; Baláš et al., 2012), we interpret that due to a higher climbing specific muscle strength, advanced climbers were able to master a greater estimated maximal reaching distance and MAC in the *Remove* condition than intermediate climbers.

In the *Move Up* condition, which also involved *more than two* nested affordances (as participants had to reach to grasp, before using this hold to transition to another hold), advanced climbers exhibited greater estimated maximal reaching distance than intermediate climbers. As already suggested in previous research, this finding appears to relate to the attunement of experts to nested information that reflects the functional properties of holds (Boschker et al., 2002), and enables experts to chain movements as they consider several holds as nested affordances (Boschker & Bakker, 2002; Seifert et al., 2017; Seifert et al., 2021). For example, various climbing action modes require specific bodily configurations with respect to orientation and the relative position of numerous holds (Seifert et al., 2015; Seifert et al., 2018). In the *Move Up* condition, multiple holds might be collectively perceived as a single, nested, climbing opportunity, which could be realized through functionally equivalent movements that advanced climbers are capable of executing (Seifert et al., 2021).

Finally, the effect of task complexity was not significant when estimated maximal reaching distance was scaled to the MAC. Thus, although the *Remove* condition exhibited the lowest estimated maximal reaching distance and MAC compared with the three other conditions, these differences were no longer significant, when estimated maximal reaching distance was scaled to MAC. Further research where the experimental setting manipulates and assesses the demands on muscular strength would contribute to better understanding of how task specificity influences the calibration of perception–action in reaching-grasping. Our findings confirmed that the performance of goal-directed behaviours, in particular nested behaviours, are action-scaled relative to task demands (Higuchi et al., 2011; Mark et al., 2015; Weast et al., 2011).

In conclusion, our findings contribute to the theory of affordances, in particular the research concerning nested affordances and calibration. First, although the theory of affordances is well established, only a relatively small number of experimental studies have examined the concept of nested affordances (often limited to two nested affordances; Mark et al., 2015; Wagman et al., 2016; Ye et al., 2009). Therefore, we addressed this gap in the literature and improved understanding on the ‘nestedness’ of affordances, through the examination of task complexity (i.e., *two* compared with *more than two* nested affordances) during climbing. We observed greater estimated maximal reaching distance and greater MAC in *two* compared with *more than two* nested affordances; however, task complexity did not influence the estimated/mastered maximal reaching distance when scaled to MAC, confirming that calibration was of equivalent accuracy, independent of task complexity.

Second, in the affordance-based control framework (Fajen, 2005), the calibration of perception–action is proposed to improve with skill level. However, when the effect of skill level has been explored in previous research, studies have not always examined calibration with reference to the maximal action capabilities of individuals. Our findings showed that advanced climbers had greater MAC but did not act nearer to their MAC than intermediate climbers. Two possible reasons that would need further investigation to examine this finding are considered (1) because of their greater MAC, advanced climbers perceive different opportunities for action than intermediate climbers, notably in the case of *more than two* nested affordances, and thus engage in different modes of action reflecting more effective chaining movements; and (2) in climbing, because the consequence of overestimation means falling and could cause injury or worse, climbers might safely scale their actions within their action boundaries to succeed and to prevent injury (Fajen, 2005). This could explain why both advanced and intermediate climbers scaled their action at the same ratio of their MAC.
